# Comparison of Nitinol Stapes Pistons with Conventional Stapes Pistons: A Cadaver Study

**DOI:** 10.5402/2011/932849

**Published:** 2011-07-06

**Authors:** Samuel A. Spear, James V. Crawford

**Affiliations:** ^1^48th Medical Group, ENT Department, Unit 5201, Box 230, Suffolk, IP279PN, UK; ^2^Division of Otolaryngology/Head and Neck Surgery, Madigan Army Medical Center, Tacoma, WA 98431-1100, USA

## Abstract

*Objective*. To visually compare the Nitinol “smart” stapes prosthesis to conventional manual crimping stapes pistons in temporal bone cadaver specimens. *Main Outcome Measures*. 10 otolaryngologists were given a photograph of the randomly ordered stapes pistons and asked to use the pictures to answer questions about each stapes piston. The answers to the survey were then recorded for analysis. *Results*. 8 of 9 Nitinol pistons were described as circular, and 3 of 9 manual crimped pistons were described as circular (*P* < .05). 6 of 9 Nitinol pistons were considered to be in contact with >66% of the incus and 3 of 9 to be in contact with 34–66% of the incus. 3 of 9 manually crimped pistons were considered to be in contact with >66% of the incus, 3 with 34–66% contact and 3 with less than 34% contact. *Conclusions*. The Nitinol “smart” stapes pistons were considered to provide a more circular and circumferential crimping and to have greater contact with the long process of the incus than conventional stapes pistons.

## 1. Introduction 

Otosclerosis is a common disorder of the bony labyrinth affecting approximately 1% of individuals in some populations. The disease is characterized by abnormal bone remodeling. Etiology is unknown but there is a well-established hereditary predisposition. The most common clinical manifestation is a conductive hearing loss as a result of stapes fixation. Surgical treatment is centered on replacement of the stapes in order to close the airbone gap and reduce the conductive hearing loss [1]. 

Since the first stapedectomy by J. Shea Jr. in 1956, there has been significant refinement of surgical methods in addition to new piston designs and materials. However, what contribution certain aspects of stapes surgery make to outcome have remained uncertain and incompletely understood, such as the manual crimping of the stapes prosthesis/piston to the incus and the subsequent incomplete resolution of their conductive hearing loss. Recurrent or persistent conductive hearing loss may require revision stapes surgery [1]. 

Studies have shown that stapes prosthesis loosening and/or displacement are the most common cause of failure, followed by incus erosion [2]. These complications may be caused by variable piston positioning and crimping force. Despite optimizing aspects of manual crimping with experience and manual dexterity, limitations still remain due to the focal, noncircumferential nature of manual crimping, the lack of visualization of the incus undersurface, and the restricted access for a crimping tool [2]. Some preliminary study results have shown that these limitations can be overcome with use of the “smart” self-crimping shape memory Nitinol stapes piston [3, 4]. It provides a technical simplification of a surgical procedure without any apparent short-term complications. This allows for reliable and consistent airbone gap closures in patients with otosclerosis [5] and with equilivent [6, 7], or better [8], hearing outcomes when compared to conventional manual crimping stapes pistons. 

In this study, we visually compare the “smart” self-crimping shape memory Nitinol stapes prosthesis to the traditional manual crimping stapes pistons in temporal bone cadaver specimens. This may help determine if the Nitinol shape-memory stapes prosthesis will provide a more concentric crimp around the incus than manually crimped stapes prosthesis. 

## 2. Materials and Methods 

This is a prospective, nonrandomized comparison study of Nitinol self-crimping versus conventional manual crimping of stapes prosthesis in temporal bone cadaver specimens. 

Using temporal bone cadaver specimens from the Madigan Army Medical Center temporal bone bank, 10 left and 10 right temporal bone specimens were randomly selected. All specimens had previously had a mastoidectomy with facial recess preformed. All specimens then underwent stapedectomy as described below. A traditional titanium manual-crimping piston was placed in 5 left and 5 right temporal bone specimens. A Nitinol shape-memory stapes prosthesis was placed in 5 left and 5 right temporal bone specimens. All of the key portions of the procedure were performed by senior author JVC in the ENT temporal bone lab equipped with a standard McGee stapes crimper, a microdrill and KTP laser. 

Surgical steps of the stapedotomy included the following: (1) endaural approach (2) elevation of a posterior tympanomeatal flap (3) access to the stapes footplate with identification of the landmarks (stapes footplate, tympanic facial nerve, pyramidal eminence) (4) separation of the incudostapedial joint (5) division of the stapes tendon and the posterior crura using the KTP laser (6) down-fracturing of the anterior crura and removal of the stapes suprastructure (7) measuring the required piston length (8) creating a rosette on the footplate using the KTP laser (9) drilling a 0.7 mm hole in the stapes footplate with a 0.7 mm microdrill (10) piston insertion and fixation using either the manual crimping devise or heat from the KTP laser and (11) repositioning of the tympanomeatal flap. 

**Figure 1:**
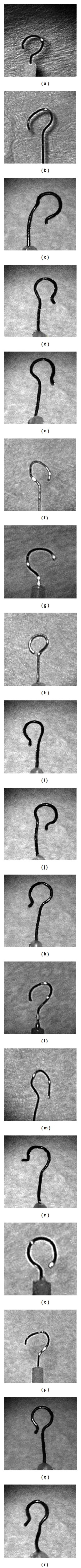
Pictures of the 18 crimped stapes pistons after dissection out of the temporal bone specimens. Conventional manual crimped pistons: (a), (b), (f), (g), (h), (l), (m), (o), and (p). Nitinol “smart” pistons: (c), (d), (e), (i), (j), (k), (n), (q), and (r).

Following completion of the stapedotomy, the incus-stapes piston unit was dissected free from the middle ear space. This was facilitated by the prior mastoid surgery. The posterior canal wall was removed as necessary to expose the incus-stapes unit. Pictures were taken of the piston after final placement transcanal. Additional pictures were taken after exposure of the unit through the mastoid, and finally pictures were taken of the incus-stapes unit ex vivo. The crimped stapes pistons were then carefully dissected free from the attached incus by dividing the incus and slipping the crimped incus slowly off without deformation of the loop. The stapes pistons were randomly assigned a letter (a–r) and photographed individually. The photographs of the pistons were matched with the in vivo and ex vivo photos and were placed on a single page for comparison (see Figure 1). 

10 otolaryngology residents and staff members were given a copy of the stapes pistons photos and asked to use the pictures to answer the following question about each stapes piston. “Would you describe this stapes piston as mostly circular (c) or oval (o)?” “Would you estimate the contact between the piston loop and the long process of the incus as: (a) <34%, (b) 34–66%, and (c) >66%?” “Do you think this is a (a) manually crimped stapes piston or (b) smart Nitinol stapes piston?” 

The answers to the survey were then recorded for analysis and results were compared with Fisher's exact test with significance of *P* <  .05. 

## 3. Results 

One Nitinol SMART stapes piston was lost while dissecting it free from the temporal bone specimen. One randomly assigned manual crimped piston was removed from the study. Survey respondents found eight out of the nine Nitinol self-crimping piston loops to be mostly circular and found three out of the nine manual crimped piston loops to be mostly circular, with the remaining to be mostly oval (see Tables 1, 2, and 3). Survey respondents found six out of the nine Nitinol self-crimped piston loops to be in contact with >66% of the long process of the incus and three out of the nine to be in contact with 34–66% of the incus. Survey respondents found three out of the nine manually crimped pistons to be in contact with >66% of the long process of the incus, three out of the nine were considered to have between 34–66% contact with the incus, and three out of the nine were considered to have less than 34% contact with the long process of the incus (see Tables 1, 2 and 3). Survey respondents could not reliably determine which crimped stapes pistons were the Nitinol self-crimped pistons or the manual crimped pistons. 

## 4. Discussion 

This is one of the first visual comparison studies of Nitinol self-crimping versus conventional manual crimping of stapes prosthesis in temporal bone cadaver specimens. Some preliminary study results have shown that use of the “smart” self-crimping shape-memory Nitinol stapes piston may eliminate drawbacks of manual crimping in stapedotomy in patients with otosclerosis, allowing more reliable and consistent airbone gap closures [4]. Studies have shown that stapes prosthesis loosening and displacement are the most common causes of failure, followed by incus erosion [2]. These complications may be caused by variable piston positioning and crimping force. 

In a study of the histopathology of the incus after stapedectomy by Gibbin [9], analysis of the morphologic changes in revision cases demonstrated that incus erosion is the result of either the abrasive friction of the piston on the bare incus that leads to resorptive osteitis with subsequent piston transmigration and tip loss, or is caused by the postoperative chemical inflammation induced by the piston material [9]. 

Despite optimizing aspects of manual crimping with experience and manual dexterity, limitations still remain due to the focal, noncircumferential nature of manual crimping, the lack of visualization of the incus undersurface, and the restricted access for a crimping tool [2]. 

Therefore, a piston that provided a more circular and circumferential crimping with greater contact with the long process of the incus should lead to less incus erosion or prosthesis loosening and displacement. 

An animal study of the vascular channels in the auditory ossicles by Anson and Winch [10] demonstrated that circumferential crimping does not lead to incus necrosis because the blood supply to the long process mainly passes through the bone marrow of the incus and to a much lesser extent through the mucoperiosteal layer [10]. 

In 1997, Kasano and Morimitsu found that use of a nickel-titanium (Nitinol) shape memory for stapes prosthesis eliminated the need for piston manipulation, and subsequently decreased macroscopic damage to the mucoperiosteum of the incus [11]. 

Studies documenting the development of the Nitinol shape memory stapes prosthesis followed by preliminary cadaver and human use results were published by Knox and Reitan in 2005 [5] and in another study by Babighian et al. in 2007 [4]. Knox and Reitan [5] described a prospective laboratory and clinical study to develop the Nitinol stapes prosthesis for use in stapedotomy surgery for otosclerosis. After development of the piston, trial surgeries were performed on human temporal bones followed by preliminary human subject trials. While human temporal bone specimens were used in this study, these were limited, and no objective data were collected [5]. Babighian et al. also studied the shape and uniformity of the Nitinol stapes loop grip in temporal bone specimens and found the loop to be uniformly surrounding the ossicle, without “dead” spaces [4]. 

Another preliminary trial in 2005 by Rajan et al. [3], outlined a prospective, preliminary case-control study involving 16 patients with otosclerosis undergoing stapedotomy using the Nitinol stapes piston. Postoperative short-term hearing results were investigated at 3, 6, and 9 months postoperatively and compared to matched reference patients who had undergone conventional titanium piston stapedotomies. Preliminary results found significantly smaller postoperative airbone gaps and interindividual variations compared to the control group suggesting that the Nitinol stapes piston eliminates the limitations of manual malcrimping in stapedotomy [3]. 

Further studies have confirmed these results. Recent published studies by Brown and Gantz [6] and Haris and Gong [7] found no statistically significant difference in hearing results when comparing the use of Nitinol stapes pistons with conventional stapes prosthesis. Huber et al. concluded that tight fixation, as provided by Nitinol prostheses, leads to improved functional results because of better sound transmission properties at the incus-prosthesis interface [8]. This study found an improvement in abg closure in the range of 3 dB pure-tone average and more pronounced at higher frequencies [8].

In this study, we visually compare the shape of traditional manually crimped stapes pistons with that of self-crimping, shape memory Nitinol stapes pistons in human temporal bone specimens with the purpose of providing confirmatory findings of recent studies that have shown elimination of drawbacks of manual crimping in stapedotomy. This provides a technical simplification of the surgical procedure with reliable and consistent airbone gap closures in patients with otosclerosis. 

Indeed, a majority of survey respondents found most of the Nitinol self-crimping piston loops (8 out of 9) to be mostly circular and only three of the nine manual crimped piston loops to be mostly circular, with the remaining to be mostly oval (see Table 3). A majority of survey respondents estimated that six out of the nine Nitinol self-crimped piston loops to be in contact with >66% of the long process of the incus while three of the nine manually crimped pistons to be in contact with >66% of the long process of the incus. In a sign that there was no selection bias, survey respondents could not reliably determine which crimped stapes pistons were the Nitinol self-crimped pistons or the manual crimped pistons. 

## 5. Conclusion 

Based on the examination of the crimped pistons and the responses of the above survey, the use of the manually crimped stapes pistons were considered more oval and less circumferential in shape with significantly less contact with the long process of the incus. This would infer more variable piston positioning and crimping force. The use of the “smart” self-crimping shape-memory Nitinol stapes piston was considered to provide a more circular and circumferential crimping and greater contact with the long process of the incus. This would infer more consistent piston positioning and crimping force. 

Use of the “smart” self-crimping shape-memory Nitinol stapes piston eliminates some of the drawbacks of manual crimping in stapedotomy and provides a technical simplification of the stapedotomy procedure in patients with otosclerosis. This should reduce stapes prosthesis loosening, displacement and incus erosion and allow for more reliable and consistent hearing results.

## Figures and Tables

**Table 1:** Survey results for the conventional manual crimped stapes prosthesis (%), *n* = 10 (survey respondents).

Piston Letter	Considered a Nitinol piston	Considered a conventional piston	Considered circular	Considered oval	Considered to have <34% contact with the incus	Considered to have 33–46% contact with the incus	Considered to have >66% contact with the incus
(a)	60%	40%	90%	10%	40%	0%	50%
(b)	30%	70%	0%	100%	10%	50%	40%
(f)	30%	70%	20%	80%	10%	60%	30%
(g)	50%	50%	40%	60%	50%	40%	10%
(h)	40%	60%	80%	20%	0%	20%	80%
(l)	30%	70%	10%	90%	60%	20%	20%
(m)	30%	70%	0%	100%	20%	50%	30%
(o)	50%	50%	90%	10%	30%	20%	50%
(p)	30%	70%	20%	80%	80%	20%	0%

**Table 2:** Survey results for the Nitinol SMART crimped stapes prosthesis (%), *n* = 10 (survey respondents).

Piston Letter	Considered a Nitinol piston	Considered a conventional piston	Considered circular	Considered oval	Considered to have <34% contact with the incus	Considered to have 33–46% contact with the incus	Considered to have >66% contact with the incus
(c)	40%	60%	40%	60%	20%	50%	30%
(d)	60%	40%	100%	0%	10%	20%	70%
(e)	50%	50%	80%	20%	10%	40%	50%
(i)	60%	40%	90%	10%	10%	20%	70%
(j)	50%	50%	80%	20%	20%	0%	80%
(k)	50%	50%	90%	10%	10%	10%	80%
(n)	50%	50%	70%	30%	40%	60%	0%
(q)	50%	50%	90%	10%	10%	30%	60%
(r)	50%	50%	90%	10%	10%	50%	40%

**Table 3:** Survey results for the Nitinol SMART crimped stapes prosthesis (%), *n* = 9 (total of each type of piston).

Piston Type	# Pistons considered circular by ≥50% of respondents, *n* = 9	# Pistons considered oval by ≥50% of respondents, *n *= 9	*P* value	# of pistons considered to have <34% contact with the incus by ≥50% of respondents	# of pistons considered to have 34–66% contact with the incus by ≥50% of respondents	# of pistons considered to have <66% contact with the incus by ≥50% of respondents	*P* value
Conventional	3 (33%)	6 (66%)	<0.05	3 (33%)	3 (33%)	3 (33%)	0.35
Nitinol	8 (88.9%)	1 (11.1%)		0	3 (33%)	6 (66%)	

## References

[1] Glasscock ME, Gulya AJ (2003). *Surgery of the Ear*.

[2] Shambaugh GE (1967). Factors influencing results in stapes surgery a long-term evaluation. *Annals of Otology, Rhinology and Laryngology*.

[3] Rajan GP, Atlas MD, Subramaniam K, Eikelboom RH (2005). Eliminating the limitations of manual crimping in stapes surgery? A preliminary trial with the shape memory nitinol stapes piston. *Laryngoscope*.

[4] Babighian G, Fontana M, Caltran S, Ciccolella M, Amadori M, De Zen M (2007). The heat-activated stapes prosthesis “SMart” Piston: technique and preliminary results. *Advances in Oto-Rhino-Laryngology*.

[5] Knox GW, Reitan H (2005). Shape-memory stapes prosthesis for otosclerosis surgery. *Laryngoscope*.

[6] Brown KD, Gantz BJ (2007). Hearing results after stapedotomy with a nitinol piston prosthesis. *Archives of Otolaryngology: Head and Neck Surgery*.

[7] Haris JP, Gong S (2007). Comparison of hearing results of nitinol SMART stapes pistion prosthesis with conventional piston prostheses: postoperative results of nitinol stapes prosthesis. *Otology and Neurotology*.

[8] Huber AM, Veraguth D, Schmid S, Roth T, Eiber A (2008). Tight stapes prosthesis fixation leads to better functional results in otosclerosis surgery. *Otology and Neurotology*.

[9] Gibbin KP (1979). The histopathology of the incus after stapedectomy. *Clinical Otolaryngology*.

[10] Anson BJ, Winch TR (1974). Vascular channels in the auditory ossicles in man. *Annals of Otology, Rhinology and Laryngology*.

[11] Kasano F, Morimitsu T (1997). Utilization of nickel-titanium shape memory alloy for stapes prosthesis. *Auris Nasus Larynx*.

